# Supplementary material for: The Use of Fecal Microbiota Transplant in Overcoming and Modulating Resistance to Anti-PD-1 Therapy in Patients with Skin Cancer
Source: Cancers (Basel). 2024 Jan 24;16(3):499. doi: 10.3390/cancers16030499 (PMC10854771; doi:10.3390/cancers16030499)
Supplement: Supplementary file 1 [file cancers-16-00499-s001.zip › cancers-2788194-supplementary.pdf]

Supplemental Data S1: Search terms utilized included 'fmt' AND 'skin cancer' and 'fmt' AND 'melanoma'

Table S1. Quality Assessment Tool for Before-After (Pre-Post) Studies With No Control Group reported scores by 2 independent reviewers.

	Duvar	Routy	Baruch	Routy	Davar	Baruch
1. Was the study question or objective clearly stated?	y	y	y	y	y	y
2. Were eligibility/selection criteria for the study population prespecified and clearly described?	y	y	y	y	y	y
3. Were the participants in the study representative of those who would be eligible for the test/service/intervention in the general or clinical population of interest?	y	y	y	y	y	y
4. Were all eligible participants that met the prespecified entry criteria enrolled?	y	y	n	y	y	n
5. Was the sample size sufficiently large to provide confidence in the findings?	n	n	n	n	n	n
6. Was the test/service/intervention clearly described and delivered consistently across the study population?	y	y	y	y	y	y
7. Were the outcome measures prespecified, clearly defined, valid, reliable, and assessed consistently across all study participants?	y	y	y	y	y	y
8. Were the people assessing the outcomes blinded to the participants' exposures/interventions?	n	n	n	n	n	n
9. Was the loss to follow-up after baseline 20% or less? Were those lost to follow-up accounted for in the analysis?	y	y	y	y	y	y
10. Did the statistical methods examine changes in outcome measures from before to after the intervention? Were statistical tests done that provided p values for the pre-to-post changes?	n	y	y	y	n	y
11. Were outcome measures of interest taken multiple times before the intervention and multiple times after the intervention (i.e., did they use an interrupted time-series design)?	y	y	y	y	y	y
12. If the intervention was conducted at a group level (e.g., a whole hospital, a community, etc.) did the statistical analysis take into account the use of individual-level data to determine effects at the group level?	n	y	y	y	n	y
**Quality Rating (Good, Fair, or Poor)**	fair	good	good	good	fair	good

Table S2. List of excluded articles.

Fecal Microbiota Transplantation for Immune Checkpoint Inhibitor-Induced Colitis Is Safe and Contributes to Recovery: Two Case Reports
First-in-class Microbial Ecosystem Therapeutic 4 (MET4) in combination with immune checkpoint inhibitors in patients with advanced solid tumors (MET4-IO trial)
Therapeutic Perspectives for Microbiota Transplantation in Digestive Diseases and Neoplasia—A Literature Review
Screening costs associated with donor selection for fecal microbiota transplantation for treatment of PD-1 refractory melanoma patients
Exploring the impact of microbiome in the response of combined radiation with immune checkpoint blockade in muscle invasive bladder cancer (MIBC)
Fecal Microbiota Transplantation as a Cancer Therapeutic
Photothermal Properties of IR-780-Based Nanoparticles Depend on Nanocarrier Design: A Comparative Study on Synthetic Liposomes and Cell Membrane and Hybrid Biomimetic Vesicles
Efficacy of fecal microbiota transplantation in patients with anti-PD-1-resistant/refractory gastrointestinal cancers

The Microbiome in Advanced Melanoma: Where Are We Now?
Fecal microbiota transplantation plus anti-PD-1 immunotherapy in advanced melanoma: a phase I trial
Fecal microbiota transplantation and its repercussions in patients with melanoma refractory to anti-PD-1 therapy: scope review
Conversion of unresponsiveness to immune checkpoint inhibition by fecal microbiota transplantation in patients with metastatic melanoma: study protocol for a randomized phase Ib/IIa trial
120TiP Conversion of response to immune checkpoint inhibition by fecal microbiota transplantation in patients with metastatic melanoma: A randomized phase Ib/IIa trial
Modulation of cancer immunotherapy by the microbiome: Insights from computational analyses of preclinical studies
Gut Microbiota and Tumor Immune Escape: A New Perspective for Improving Tumor Immunotherapy
Onco-biome in pharmacotherapy for lung cancer: a narrative review
MICROBIOME MODIFICATION WITH FECAL MICROBIOTA TRANSPLANT FROM HEALTHY DONORS BEFORE ANTIPD1 THERAPY REDUCES PRIMARY RESISTANCE TO IMMUNOTHERAPY IN ADVANCED AND METASTATIC MELANOMA PATIENTS
Intestinal Microbiota: The Driving Force behind Advances in Cancer Immunotherapy
Gut Microbiota and Therapy in Metastatic Melanoma: Focus on MAPK Pathway Inhibition
DONOR MICROBIAL ENGRAFMENT AFTER FECAL MICROBIOTA TRANSPLANTATION: CHARACTERISTICS AND ASSOCIATION WITH CLINICAL RESPONSE ACROSS DIFFERENT DISORDERS
Fecal Transplantation Improved Patients' Reported Outcome After Immune Checkpoint Inhibitor Colitis
LONGITUDINAL CHANGES IN THE GUT MICROBIOME IN RESPONSE TO IMMUNE CHECKPOINT BLOCKADE
Treatment of Refractory ICI-Associated Colitis With Fecal Microbiota Transplant
IL12/23 Blockade for Refractory Immune-Mediated Colitis: A Case Series From Two Centers
Antitumor effects of fecal microbiota transplantation: Implications for microbiome modulation in cancer treatment
Using the canine microbiome to bridge translation of cancer immunotherapy from pre-clinical murine models to human clinical trials
Dermatofibrosarcoma protuberans of the hallux: A case report with review of the literature
Fecal Microbiota Transplantation Effectively Cures a Patient With Severe Bleeding Immune Checkpoint Inhibitor-Associated Colitis and a Short Review
Fecal microbiota transplantation followed by anti-PD-1 treatment in patients with advanced melanoma.
Could fecal microbial transplantation offer a new potential in the treatment of metastatic pancreatic ductal adenocarcinoma?
Optimizing therapeutic outcomes of immune checkpoint blockade by a microbial tryptophan metabolite
The impact of the microbiome on resistance to cancer treatment with chemotherapeutic agents and immunotherapy
Cytotoxic Activity and Antibiofilm Efficacy of Biosynthesized Silver Nanoparticles against Methicillin-Resistant Staphylococcus aureus Strains Colonizing Cell Phones
Fecal microbiota transplantation – new possibility to influence the results of therapy of cancer patients
Combining Fecal Microbiota Transplantation with Immunotherapy in Treatment-Naive Patients with Advanced Melanoma
Gut microbiota shift in melanoma patients undergoing immunotherapy is associated with clinical response
Fecal microbiota transplantation to improve efficacy of immune checkpoint inhibitors in renal cell carcinoma (TACITO trial)
Incongruence between dominant commensal donor microbes in recipient feces post fecal transplant and response to anti-PD-1 immunotherapy

Steroid-resistant intestinal aGVHD and refractory CMV and EBV infections complicated by haplo-HSCT were successfully rescued by FMT and CTL infusion
FMT for patients with cancer
Gut Microbiota Tuning Promotes Tumor-Associated Antigen Cross Presentation and Enhances CAR T Antitumor Effects
The gut microbiome and cancer immunotherapy: Can we use the gut microbiome as a predictive biomarker for clinical response in cancer immunotherapy?
IL12/23 blockade therapy for refractory immune checkpoint inhibitor colitis: A case series
Fecal microbiota transplantation and fiber supplementation, better together?
The Challenge of ICIs Resistance in Solid Tumours: Could Microbiota and Its Diversity Be Our Secret Weapon?
Intestinal microbiota: A potential target for enhancing the antitumor efficacy and reducing the toxicity of immune checkpoint inhibitors
Efficacy of Responder-derived Fecal Microbiota Transplant (R-FMT) and Pembrolizumab in Anti-PD-1 Refractory Patients with Advanced Melanoma
First clinical proof-of-concept that FMT can overcome resistance to ICIs
Successful treatment with paclitaxel of a visceral relapse of post-transplant Kaposi's sarcoma
A multilobulated asymptomatic umbilical nodule revealing endometriosis
Fecal microbiota transplant overcomes resistance to anti-PD-1 therapy in melanoma patients
Fecal microbiota transplant promotes response in immunotherapy-refractory melanoma patients
FMT for immunotherapy-refractory melanoma
Ferumoxytol-β-glucan inhibits melanoma growth via interacting with dectin-1 to polarize macrophages into m1 phenotype
Targeting and pharmacology of an anti-IL13Rα2 antibody and antibody-drug conjugate in a melanoma xenograft model
Safety and efficacy of fecal microbiota transplantation to treat and prevent recurrent Clostridioides difficile in cancer patients
Association between the gut microbiota and patient responses to cancer immune checkpoint inhibitors (Review)
Fecal Microbiota Transplant (FMT) for Immune Checkpoint Inhibitor (ICI) Induced-Colitis (IMC) Refractory to Immunosuppressive Therapy
The effect of intestinal flora on immune checkpoint inhibitors in tumor treatment: A narrative review
Immunotherapy in Colorectal Cancer: Potential of Fecal Transplant and Microbiota-Augmented Clinical Trials
Gut Microbiome Modulation Via Fecal Microbiota Transplant to Augment Immunotherapy in Patients with Melanoma or Other Cancers
Intestinal microbiota: A new force in cancer immunotherapy
Combination of fecal microbiota transplantation from healthy donors with anti-pd1 immunotherapy in treatment-native advanced or metastatic melanoma patients
Leveraging gut microbiota networks to impact tumor immunotherapy
Gut microbiota regulate tumor metastasis via circRNA/miRNA networks
Fecal microbiota transplantation (FMT) for immune checkpoint inhibitor induced-colitis (IMC) refractory to immunosuppressive therapy
Fecal microbiota transplantation in cancer management: Current status and perspectives
Fecal microbiota transplantation in metastatic melanoma patients resistant to anti-PD-1 treatment
Fecal microbiota transplantation (FMT) and re-induction of anti-PD-1 therapy in refractory metastatic melanoma patients - Preliminary results from a phase I clinical trial (NCT03353402)

Leveraging gut microbiota networks to impact tumor immunotherapy
Stool donor qualification and collection from metastatic melanoma (MM) patients (PTS) who have responded to checkpoint inhibitors (CPI) for manufacturing of fecal microbiota transplantation (FMT)
Manipulating the gut microbiome to improve immunotherapy of melanoma
Leveraging gut microbiota networks to impact tumor immunotherapy
Impact of the gut microbiota on immune checkpoint inhibitor-associated toxicities
Preliminary results from a microbiome-based phase i clinical trial - Fecal microbiota transplantation in metastatic melanoma patients who failed immunotherapy (NCT03353402)
Compositional differences in the gut microbiome are associated with distinct tumor and systemic immune profiles in melanoma
Patient-derived microbiota germ-free mouse model for identifyingmechanisms of checkpoint blockade efficacy modulation
Characteristics of the microbiome form and function of complete responders to Anti-PD1 and healthy individuals implicate donor selection for clinical trial design
Variation of the gut microbiome of complete responders to immune checkpoint blockade and healthy individuals-implications for clinical trial design
Anti-tumor macrophages activated by ferumoxytol combined or surface-functionalized with the TLR3 agonist poly (I: C) promote melanoma regression
Diversity and composition of the gut microbiome influences responses to anti-PD1-therapy through beneficial changes in innate and adaptive immunity
A novel CXCR4-targeted near-infrared (NIR) fluorescent probe (Peptide R-NIR750) specifically detects CXCR4 expressing tumors
Comparison of FMT/Micro-CT with Micro-PET/MRI for monitoring pharmacokinetics and biodistribution of different antibody formats in a human skin cancer xenograft model
In vivo imaging of CXCR4 expressing human cancer cells with a novel Near-Infrared labelled-CXCR4 cyclic peptide antagonist Peptide R
Ipilimumab-associated colitis or refractory Clostridium difficile infection?
Immune monitoring in cancer vaccine clinical trials: Critical issues of functional flow cytometry-based assays
Imaging of tumor vascularization using fluorescence molecular tomography to monitor arginine deiminase treatment in melanoma.
Preclinical evaluation of a novel therapeutic approach in malignant melanoma using micropet and fluorescence molecular tomography
Sarcoidosis and sarcoid reactions in cancer
In vivo imaging of integrin $\alpha v\beta 3$ expression using fluorescence-mediated tomography
Screening HIV-positive pregnant women for antiretroviral therapy: Utility of self-reported symptoms
Kaposi's sarcoma-associated immune reconstitution following highly active antiretroviral treatment initiation [4]
National trials involving lymphatic mapping for melanoma: The multicenter selective lymphadenectomy trial, the SunBelt melanoma trial, and the Florida melanoma trial
Fecal microbiota transplant promotes response in immunotherapy-refractory melanoma patients.
Fecal microbiota transplant overcomes resistance to anti-PD-1 therapy in melanoma patients
Intestinal dysbiosis exacerbates the pathogenesis of psoriasis-like phenotype through changes in fatty acid metabolism
[Sarcoidosis and sarcoid reactions in cancer]
Fecal Microbiota Transplant in Immunotherapy-Resistant Melanoma: What Can We Expect in the Near Future?

Functional Muscle Transfer after Oncologic Extremity Resection
Ferumoxytol-β-glucan Inhibits Melanoma Growth via Interacting with Dectin-1 to Polarize Macrophages into M1 Phenotype
Gut microbiota mediated the toxicity of high concentration of dietary nitrite in C57BL/6 mice
National trials involving lymphatic mapping for melanoma: the Multicenter Selective Lymphadenectomy Trial, the Sunbelt Melanoma Trial, and the Florida Melanoma Trial
Conversion of unresponsiveness to immune checkpoint inhibition by fecal microbiota transplantation in patients with metastatic melanoma: study protocol for a randomized phase Ib/IIa trial
A novel CXCR4-targeted near-infrared (NIR) fluorescent probe (Peptide R-NIR750) specifically detects CXCR4 expressing tumors
Fecal microbiota transplant promotes response in immunotherapy-refractory melanoma patients
Commensal Bifidobacterium promotes antitumor immunity and facilitates anti-PD-L1 efficacy.
Gut microbiome modulates response to anti-PD-1 immunotherapy in melanoma patients
Anticancer immunotherapy by CTLA-4 blockade relies on the gut microbiota.
The microbiota and immune-mediated diseases: Opportunities for therapeutic intervention.
The emerging potential of microbiome transplantation on human health interventions.
The cure within? a review of the bicrobiome and diet in melanoma
Differential diagnosis and management of immune checkpoint inhibitor-induced colitis: A comprehensive review.
Universality of human micorbial dynamics
MAVS deficiency induces gut dysbiotic microbiota conferring a proallergic phenotype.
Screening costs associated with donor selection for fecal microbiota transplantation for treatment of PD-1 refractory melanoma patients.
Intestinal decontamination of multidrug-resistant Klebsiella pneumoniae after recurrent infections in an immunocompromised host.

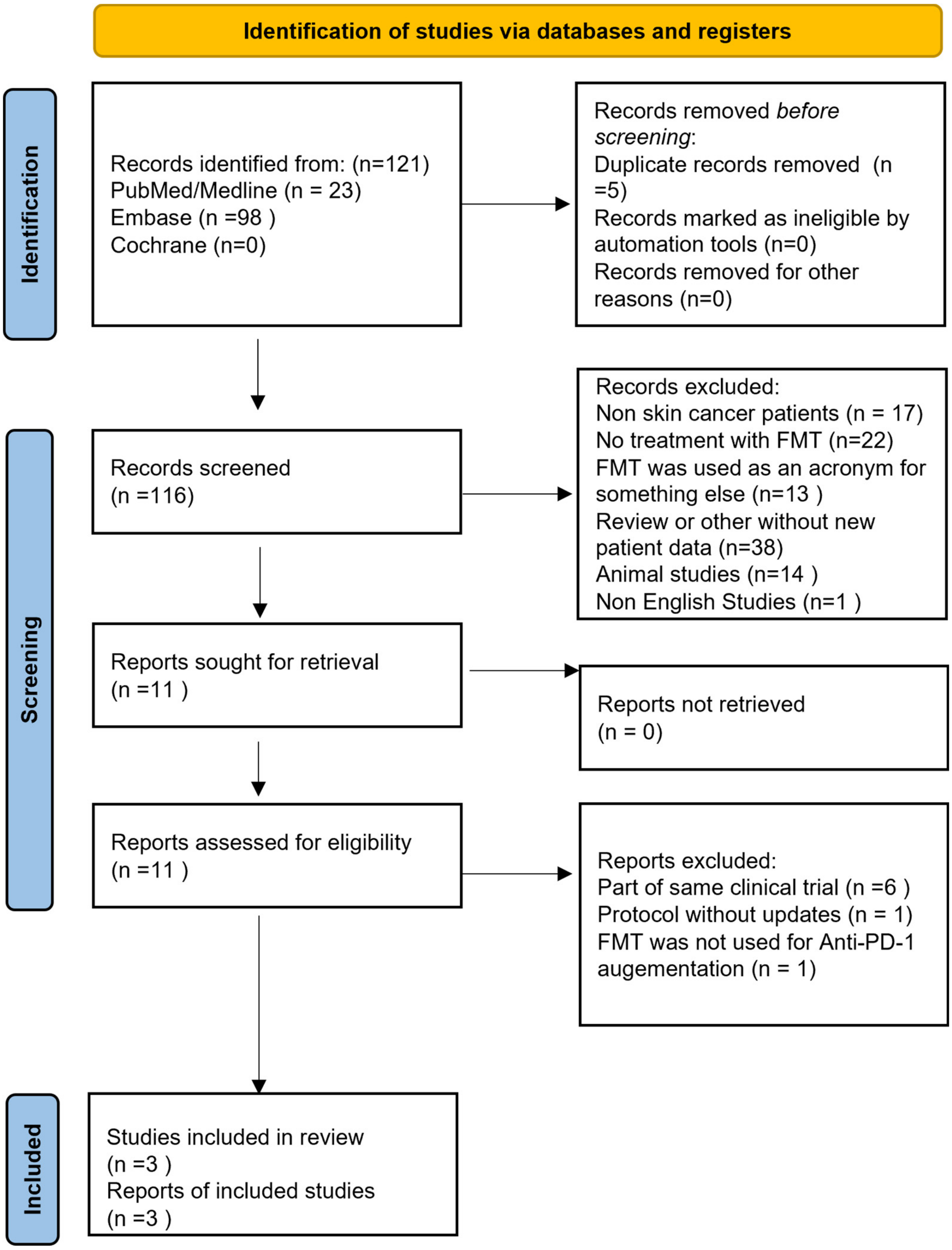

Figure S1. PRISMA 2020 flow diagram for new systematic reviews which included searches of databases and registers only. *Consider, if feasible to do so, reporting the number of records identified from each database or register searched (rather than the total number across all databases/registers). **If automation tools were used, indicate how many records were excluded by a human and how many were excluded by automation tools. *From:* Page M.J. et al. [21].

For more information, visit: http://www.prisma-statement.org/
